# Deciphering the active constituents of Dabushen decoction of ameliorating osteoarthritis *via* PPARγ preservation by targeting DNMT1

**DOI:** 10.3389/fphar.2022.993498

**Published:** 2022-11-23

**Authors:** Lu Qiu, Min Zhang, Chenghao Li, Yehu Hou, Hao Liu, Jia Lin, Juan Yao, Dong Zhu Duan, Yi Xi Zhang, Mi Li, Ya Ling Li, Peng Wang, Jin Tian Li, Xiao Jie Jin, Yong Qi Liu

**Affiliations:** ^1^ Gansu University Key Laboratory for Molecular Medicine and Chinese Medicine Prevention and Treatment of Major Diseases, Gansu University of Chinese Medicine, Lanzhou, China; ^2^ Key Laboratory of Dunhuang Medicine, Ministry of Education, Gansu University of Chinese Medicine, Lanzhou, China; ^3^ College of Pharmacy, Gansu University of Chinese Medicine, Lanzhou, China; ^4^ Shaanxi Key Laboratory of Phytochemistry and College of Chemistry and Chemical Engineering, Baoji University of Arts and Sciences, Baoji, China

**Keywords:** traditional Chinese medicine, Dabushen decoction, osteoarthritis, epigenetics, Dnmt1, PPARγ

## Abstract

Osteoarthritis (OA) is a multifactorial and chronic degenerative joint disease. Due to the adverse effects of currently used drugs, a safer and more effective therapy for treating OA is needed. Peroxisome proliferator-activated receptor-γ (PPARγ) is a key protein protecting cartilage. DNMT1-mediated hypermethylation of PPARγ promoter leads to its suppression. Therefore, DNMT1 might be an effective target for exerting cartilage protective effects by regulating the epigenetic expression of PPARγ. Dabushen decoction (DD) is a representative prescription of Dunhuang ancient medical prescription, which has a potential therapeutic effect on OA. So far, the research of the efficacy and material basis of DD in the treatment of OA remains unclear. In this study, Micro-CT, HE staining, S-O staining, and immunohistochemistry analysis were used to demonstrate that DD increased the expression of PPARγ and collagen synthesis in an OA rat model. Next, the structure of DNMT1 was used to screen the active constituents of DD by molecular docking method for treatment OA. Seven potential active constituents, including isoliquiritigenin, emodin, taxifolin, catalpol, alisol A, zingerone, and schisandrin C were hited. The protective effect of the potential active constituents to chondrocytes were evaluated by protein capillary electrophoresis, immunofluorescence assays, and *ex vivo* culture of rat knee cartilage. The five constituents, such as alisol A, emodin, taxifolin, isoliquiritigenin, and schisandrin C could promote the expression of PPARγ and ameliorate IL-1β-induced downregulation of collagen II and the production of MMP-13. Alisol A and Emodin could effectively mitigate cartilage damage. At last, molecular dynamics simulations with MM-GBSA method was applied to investigate the interaction pattern of the active constituents and DNMT1 complexes. The five constituents, such as alisol A, emodin, taxifolin, isoliquiritigenin, and schisandrin C achieved a stable binding pattern with DNMT1, in which alisol A has a relatively high binding free energy. In conclusion, this study elucidates that the active constituents of DD (alisol A, emodin, taxifolin, isoliquiritigenin, and schisandrin C) could ameliorate osteoarthritis *via* PPARγ preservation by targeting DNMT1.These findings facilitated clinical use of DD and provided a valuable strategy for developing natural epigenetic modulators from Chinese herbal formula.

## Introduction

Osteoarthritis (OA), a disease of joint degeneration characterized by articular cartilage (AC) loss, often causes pain, swelling, stiffness, and joint deformation (Pigeolet et al., 2021). It is a multifactorial disease that can be caused by injury, genetics, age, obesity and gender, etc (Wang et al., 2011). Numerous studies have suggested that the pathogenesis of OA mainly involves inflammatory reactions, metabolic disorders, and imbalanced oxidative stress (Barter et al., 2012; Robinson et al., 2016; Dickson et al., 2019; Ansari et al., 2020). Pharmacologic therapy of OA is mainly aimed at pain relief, including non-steroidal anti-inflammatory drugs and opioids (Abramoff and Caldera, 2020; D'Arcy et al., 2021; da Costa et al., 2021). However, the adverse effects of these drugs, including hepato and nephrotoxicity, loss of gastrointestinal motility and physical and psychological dependence emphasize the need to develop a safer and more effective therapy for treating OA.

A reported protective role for peroxisome proliferator-activated receptor-γ (PPARγ) in OA raises the possibility that upregulation of PPARγ may be beneficial in the context of preventing and treating OA (Afif et al., 2007). PPARγ is a member of ligand-activated nuclear transcription factor family and plays an essential role in cartilage health; the lack of PPARγ accelerates the onset of spontaneous OA (Vasheghani et al., 2015). PPARγ is expressed and functionally active in chondrocytes (Afif et al., 2007). Research has shown that PPARγ activators display anti-inflammatory and chondroprotective properties *in vitro* and improve the clinical course and histopathological features in an experimental animal model of OA (Bordji et al., 2000; Kobayashi et al., 2005; Li et al., 2016). Therefore, increasing the expression of PPARγ in cartilage might be an effective strategy to slow or prevent OA progression.

Recent studies have clearly shown that aberrant DNA methylation can lead to PPARγ suppression (Zhu et al., 2019). DNA methylation reactions are catalyzed by the DNA methyltransferase family of enzymes (DNMT1, DNMT3A and DNMT3B) (Grandi and Bhutani, 2020; Chen et al., 2021a). DNMT1 is a maintenance methyltransferase, copying methylation patterns after DNA replication (Foulks et al., 2012). It is reported that PPARγ suppression in OA cartilage can be caused by aberrantly induced DNMT1 activity and the associated promoter hypermethylation (Zhu et al., 2019). This suggests that DNMT1 might be a valuable target for protecting and preserving PPARγ activity in OA.

Traditional Chinese medicine (TCM) has been used to treat OA for many years (Lai et al., 2015; Zhang et al., 2015; Yao et al., 2021; Sun et al., 2022). “Kidney Governing Bone” is a classical theory in TCM (Jiang et al., 2022). According to TCM syndromes, modifying kidney function is regarded as the one of the main treatments for OA. Clinical studies have demonstrated that stimulating kidney function has a beneficial effect in the therapy of OA (Sun et al., 2022). Experimental studies have shown that kidney stimulant prescriptions have beneficial effects on OA animals (Gao et al., 2012; Yuan et al., 2012). It was reported that decreased collagen synthesis resulted in the degeneration of articular cartilage of the knee in several kidney deficiency rat models (Yao et al., 2013). Dabushen decoction (DD) of Dunhuang ancient medical prescription is made up of seven Chinese herbs: *Rehmanniae radix praeparata*, *Lophatheri herba*, *Schisandrae chinensis fructus*, *Zingiberis rhizoma*, *Cinnamomi ramulus*, *Alismatis rhizoma*, *Glycyrrhizae radix et rhizoma*. In TCM, the main function of DD is to stimulate kidney function and strengthen bones, which is believed to play an important role in the treatment of OA. However, there are few studies on the protective effect of DD *in vivo*. In addition, the active constituents of DD for the treatment of OA remain unclear. In this study we first established a rat model of OA to evaluate the efficacy of DD in the treatment of osteoarthritis. Based on the effectiveness, the target DNMT1 was selected to identify the active constituents of DD in ameliorating osteoarthritis, presumably by preservation of PPARγ expression. Our results provide data for the scientific formulation of DD for the treatment of OA and provide a way for the discovery of natural epigenetic modulators from Chinese herbal formula.

## Materials and methods

### Chemicals and reagents

*Rehmanniae radix praeparata*, *Lophatheri herba*, *Schisandra chinensis Baill*, *Zingiberis rhizoma*, *Cinnamomi ramulus*, *Alismatis rhizoma* and *Glycyrrhizae radix et rhizoma* were obtained from affiliated Hospital of Gansu University of Chinese Medicine (Lanzhou, China).

Taxifolin, emodin, isoliquiritigenin, schisandrin C, and alisol A were purchased from PUSH (Chengdu, China). Catalpol and zingerone were purchased from MedChemExpress (NJ, United States). SGI-1027 was purchased from TOPSCIENCE (Shanghai, China). Purity of all reference standards was greater than 98%.

Papain and L-cysteine were procured from Sigma (St. Louis, United States). Fetal bovine serum (FBS) and Dulbecco’s Modified Eagle’s Medium F12 (DMEM/F12) were acquired from Gibco (NY, United States). Primary antibodies against GAPDH were purchased from Proteintech Group (Wuhan, China) and against collagen II, MMP-13 and PPARγ were purchased from Abcam (Cambridge, United Kingdom). ProteinSimple 042–206 was purchased from Bio-Techne (Minneapolis, United States). Enzyme-linked immunosorbent assay (ELISA) kits to detect prostaglandin E2 (PGE2) and tumour necrosis factor alpha (TNF-α) were purchased from Elabscience (Wuhan, China). 4% paraformaldehyde, EDTA decalcifying solution, 0.25% trypsin digestion solutions, collagenase, toluidine blue O solution, modified Safranine O-Fast Green FCF Cartilage Stain kit, RIPA buffer, penicillin-streptomycin liquid, HE Staining kit, 50× TAE Buffer, DMSO and Mayer’s Hematoxylin solution were procured from Solarbio (Beijing, China). Griess reagent was purchased from Beyotime Institute of Biotechnology (Shanghai, China). 3,3′-diaminobenzidine was purchased from BOSTER (Wuhan, China). MaxVision HRP-Polymer anti-Mouse IHC kit was purchased from MXB (Fuzhou, China). Cell counting kit-8 (CCK-8) and DAPI Fluoromount-G were purchased from Meilunbio (Dalian, China). Tissue/cell DNA extraction reagent kit was purchased from Bioteke (Wuxi, China). DNA Bisulfite Conversion kit, Methylation-specific PCR (MSP) kit and Marker (DNA) were purchased from Tiangen Biotech (Beijing, China). Agarose was purchased from YE SEN (Shanghai, China). GelstainRed was purchased from ACRO (Beijing, China). Loading buffer (DNA) was purchased from Vazyme (Nanjing, China).

### Rat OA model and drug administration

All experiments involving animals were approved by the Ethics Committee of Laboratory Animal Center of Gansu University of Chinese Medicine (Lanzhou, China). A total of 27 6-week-old female Specific Pathogen Free (SPF) Sprague-Dawley rats (200 ± 20 g) were used for the *in vivo* experiment (9 rats for each group). All animals were housed in a clean vivarium (specific pathogen free) at room temperature and allowed free access to standard pelleted forage and tap water. All rats were fed for 1 week for acclimatization before experiments.

DD was purchased from Affiliated Hospital of Gansu University of Chinese Medicine (Lanzhou, China). Rats were randomly divided into three groups: control, OA, DD-treated. On days 1, 4 and 7, the double knee joints of OA and DD-treated groups were injected with 0.2 ml 4% papain with L-cysteine, and double knee joints of control group were injected with an equal volume of sterile saline solution.

DD was purchased from Affiliated Hospital of Gansu University of Chinese Medicine (Lanzhou, China). DD-treated group rats with induced OA received 5.85 g/kg of DD (1 ml/100 g weight) orally. Rats in the control and OA groups were treated with an equal volume of Physiological saline. All groups were treated once a day for 6 consecutive weeks. Serum was collected from rats in all groups after anesthesia, and the knees were harvested after sacrifice.

## Micro-CT analysis for knee joints

At week 6 post-treatment the fixed knee samples were scanned by a high resolution Micro-CT (Micro-CT 80, SCANCO MEDICAL AG, Switzerland, Voltage = 55 kV, Current = 145 μA, Voxel size = 11.6 μm). We defined the region of interest (ROI) to cover the whole subchondral bone in tibial plateaus. Built-in software in the Micro-CT system was used for three-dimensional reconstruction. Bone volume/tissue volume (BV/TV) of tibial subchondral bone was evaluated in the Micro-CT system (Chen et al., 2019).

### Histological analysis

Knee joint samples were fixed in 4% paraformaldehyde for 24 h and decalcified in 10% EDTA solution for 4 weeks, after which the samples were dehydrated by graded alcohol, clarified, and embedded in paraffin blocks. Sections were stained with Safranin-O-Fast green (S-O) and Hematoxylin-eosin (HE). The Osteoarthritis Research Society International (OARSI) scoring system was used to evaluate the degree of pathological cartilage damage. All sections were scored by two experienced observers blinded to the study.

### Immunohistochemistry analysis

Immunohistochemistry sections were deparaffinized and rehydrated using a graded ethanol series and incubated overnight at 4°C with antibodies to PPARγ (1:100), collagen II (1:500), and MMP-13 (1:200). The slides were then treated with secondary antibodies, biotinylated anti-mouse IgG, and then streptavidin peroxidase complex (MXB, Fuzhou, China) for 10 min, and then 3,3′-diaminobenzidine (BOSTER, Wuhan, China) was added. The slides were counterstained with Mayer’s hematoxylin and photographed under a photomicroscope (Olympus, Tokyo, Japan). The percentage of positivity of each immunohistochemical assay was measured using ImageJ software (NIH, Bethesda, MD). Five representative images were selected for each donor (*n* = 3) and used for relative quantification. The negative controls staining is shown in Supplementary Figure S1.

### Determination of cytokines levels in serum

Sandwich enzyme-linked immunosorbent assay (ELISA) kits were used to detect the levels of inflammatory factors IL-1β, PGE2, and TNF-α in the serum of each group of rats. The ELISA procedure was performed according to the manufacturer’s instructions. The NO level in serum was detected by the Griess reagent.

## Constituents of Dabushen decoction

The structures of DD^’^s constituents were collected from the Traditional Chinese Medicines Systems Pharmacology (TCMSP, http://tcmspw.com/) (Ru et al., 2014) and Traditional Chinese Medicines Integrated Database (TCMID, http://www.megabionet.org/tcmid/) (Chen et al., 2006). Constituents of each herb were identified through a literature search in PubMed database (https://pubmed.ncbi.nlm.nih.gov/). A total of 906 constituents were retrieved for 7 herbs, including *Rehmanniae radix praeparata* (76), *Lophatheri herba* (4), *Schisandrae chinensis fructus* (129), *Zingiberis rhizoma* (148), *Cinnamomi ramulus* (221), *Atractylodes lancea* (49), *Glycyrrhizae radix et rhizoma* (279).

### Molecular docking

Molecular docking was carried out using the Glide module with the standard docking precision method (Glide SP) in Schrӧ; dinger 2020—4 (Friesner et al., 2006). Ligands were then preprocessed by LigPrep (MMFFs force fields) to generate the 3D structures and the energies of the generated conformations were minimized. The ionized states of the ligands were assigned *via* Epik at the pH value of 7.0 ± 2.0. The crystallographic structure of DNMT1 was obtained from Protein Data Bank (PDB ID: 3PTA (Hou et al., 2011)). Missing amino acids of the DNMT1 (PDB ID: 3PTA (Hou et al., 2011)) were added by method of the multiple templates homology modelling in the web server of SWISS-MODEL database (Waterhouse et al., 2018). The prepared structure was processed by Protein Preparation Wizard module in Maestro before calculating the docking grid, including adding hydrogen atoms and assigning protonation states and partial charges with OPLS_2005 force field. The pocket for docking was generated using the Receptor Grid Generation module by defining the S-adenosyl-l-homocysteine (SAH) as the center of the grid (Krishna et al., 2017).

## Isolation, culture of chondrocytes

Three-week-old Sprague-Dawley (SD) rats were euthanized and immersed in 75% medicinal alcohol for 15 min (Jiao et al., 2013). Cartilage collected from knee joints was minced and digested with 0.25% trypsin for 30 min (Tu et al., 2019b). Cartilage fragments were fully digested with 0.2% Collagenase for 4 h. Subsequently, cell suspensions were centrifuged (1,500 r/min, 5 min) to harvest primary chondrocytes. The isolated cells were resuspended in DMEM/F12 supplemented with 10% FBS and 1% penicillin/streptomycin solution at 37°C with 5% CO_2_. Toluidine blue staining and Collagenase immunofluorescence staining were utilized to identify primary rat chondrocytes. To maintain stability of the cell phenotype, the second- and third-generation cells were routinely selected for subsequent cell experiments (Tu et al., 2019a).

### Cell viability analysis

The cell viability of chondrocytes was measured by the CCK-8 assay according to the manufacturer’s protocol. Briefly, the chondrocytes were seeded into 96-well plates (5 × 10^3^ cells/well) followed by treatment with different concentrations (0, 5, 10, 20, 40, 80 μM) of isoliquiritigenin, emodin, taxifolin, catalpol, alisol A, zingerone, and schisandrin C for 24 h. At the indicated time, 100 µL of DMEM/F12 containing 10 µL of CCK-8 solution was added to each well, and the cells were then incubated for another 2 h at 37°C. The absorbance of the wells was read at 450 nm by microplate reader (Bio-Rad, Hercules, CA).

### Protein capillary electrophoresis

For capillary electrophoresis, cells were lysed directly in the Laemmli buffer (Solarbio, Beijing, China) containing 1× protease and phosphatase inhibitor cocktail (Solarbio, Beijing, China). For protein capillary electrophoresis, the primary antibodies were used rabbit anti-PPARγ (Cambridge, United States); the secondary antibodies were used with the manufacturer’s instructions: Anti-Rabbit Secondary HRP Antibody (ProteinSimple 042–206).

### Immunofluorescence assays

Chondrocytes were seeded in 24-well plates at a density of 1 × 10^4^ cells/well. Cells were cultured on coverslips and treated with 10 ng/ml IL-1β and with different concentrations of isoliquiritigenin, emodin, taxifolin, catalpol, alisol A, zingerone and schisandrin C for 24 h. Coverslips were washed blocked for 30 min after fixation, and incubated overnight at 4°C with the primary antibodies MMP-13, PPARγ, and collagen II. Incubation with Alexa Fluor 647-conjugated donkey anti-rabbit secondary antibodies was performed for 2 h at room temperature. The nuclei were counterstained with DAPI fluorescent probes and analyzed with a high content analysis system (PerkinElmer, Operetta CLS).

### ELISA and griess reaction

The concentration of TNF-α and PGE2 in cell culture supernatants was determined by using commercial ELISA kits (Elabscience, wuhan) according to the manufacturer’s instructions. The NO level in the culture medium was detected by the Griess reagent.

### *Ex Vivo* evaluation by organ culture of rat knee cartilage

Cartilage explants were harvested from the bilateral knee joints of 3-week-old SD rats. The explants were initially cultured at 37°C with 5% CO_2_ in DMEM/F12 containing 10% FBS and 1% penicillin/streptomycin solution for 2 days. Cartilage explants then were transferred to 48-well plates and cultured in serum-free medium containing 10 ng/ml IL-1β with different concentrations of constituents for 3 days (*n* = 6 explants per condition) (Latourte et al., 2017). Subsequently, explants were collected, fixed in 4% paraformaldehyde and embedded in paraffin. Samples were cut along the coronal plane and stained with HE for cartilage surface observation and Safranin O staining for degradation of cartilage matrix (Headland et al., 2015).

### Molecular dynamics simulation

The initial complex structures of the DNMT1-isoliquiritigenin, DNMT1-emodin, DNMT1-taxifolin, DNMT1-catalpol, DNMT1-alisol A, DNMT1-zingerone, and DNMT1-schisandrin C complexes were obtained from molecular docking (Yang et al.) Structure optimization and frequency calculations for the above ligand compounds were computed by Gaussian16 at the level HF/6-31G*. The restrained electrostatic potential was used to describe the partial atomic charges. The general AMBER force field (GAFF) (Wang et al., 2004), the ff14SB force field (Maier et al., 2015) and the Zinc AMBER force field (ZAFF) (Peters et al., 2010) were used for describing the active constituents, proteins and Zn^2+^, respectively. All systems were solvated in an atomistic TIP3P water box with 15 Å distance around solutes. After adding chloride ions to neutralize the systems, the systems were minimized, heated, and equilibrated. MD simulations (50 ns) were performed at 300 K with 1.0 atmospheric pressure in an NPT ensemble. The trajectory analysis was performed *via* the cpptraj module in AMBER 20 (Case et al., 2005).

#### Binding free energy calculation

The binding free energies (ΔG_bind_) of complexes were calculated *via* the endpoint molecular mechanics generalized Born surface area (MM/GBSA) approach using the following equation Eq. 1, 3: (Hou et al., 2011; Chen et al., 2016)

$${∆G}_{bind}={∆E}_{vdw}+{∆E}_{ele}+{∆G}_{pol}+{∆G}_{nonpol}$$ (1)

$${∆G}_{gas}={∆E}_{vdw}+{∆E}_{ele}+{∆E}_{\mathrm{int}},$$ (2)

$${∆G}_{solv}={∆G}_{pol}+{∆G}_{nonpol}.$$ (3)

Furthermore, we decomposed the total binding free energy into each residue by Eq. 4 to identify the key residues responsible for the binding of constituents.

$${\Delta G}_{calc}^{per-residue}={\Delta E}_{vdW}^{per-residue}+{\Delta E}_{ele}^{per-residue}+{\Delta G}_{pol}^{per-residue}+{\Delta G}_{nonpol}^{per-residue}.$$ (4)

We used 200 snapshots extracted from the last 10 ns trajectories of the system to calculate binding free energy. Residue free energy decomposition was performed to identify the key residues responsible for ligand binding by splitting the total free energy into the energy contributions from individual residue-ligand pairs.

#### Methylation-specific PCR (MSP)

DNA extraction from human chondrocytic SW1353 cells was performed with a tissue/cell DNA extraction reagent kit (Bioteke, China) according to the manufacturer’s instructions. Bisulfite modification of DNA was carried out using the DNA Bisulfite Conversion Kit (Tiangen Biotech, China) following the manufacturer’s instructions. Primers used to amplify a bisulfite converted DNA fragment as follow: Methylated primer TTA​ATT​TAT​TTT​GGA​TAG​GTT​ACG​A (forward) and TCT​TAA​CAA​TAT​TTC​TAA​CAC​CGA​A (reverse); unmethylated primer TTA​ATT​TAT​TTT​GGA​TAG​GTT​ATG​A (forward) and TTA​AAT​TTC​TTA​ACA​ATA​TTT​CTA​ACA​CCA (reverse), which amplify the –232/–76 locus. For internal control (Input), the genomic DNA was PCR amplified simultaneously with forward primer TAG​TTT​TAG​GAA​GGT​AAA​GGG​AGT​G and reverse primer AAA​TCC​CAA​AAA​AAA​CAC​AAC​AAA. The PCR reaction was performed with a Methylation-specific PCR (MSP) kit (Tiangen Biotech, China) and confirmed by electrophoresis in a 2% agarose gel, visualized under ultraviolet light, and densitometry analysis of each PCR band was analyzed using the Image J program. The relative levels of PCR products were first normalized with input PCR and then presented as ratio of methylated or unmethylated PCR over total PCR products.

### Statistical analysis

All data are expressed as the mean ± SD for each group. One-way analysis of variance (ANOVA) followed by LSD post hoc test was used to compare the significance of the group differences. Comparisons were considered significant for *p* < 0.05. Each experiment consisted of at least three replicates per condition.

## Results and disussion

### DD alleviated tibial subchondral bone and cartilage damage

**FIGURE 1:**
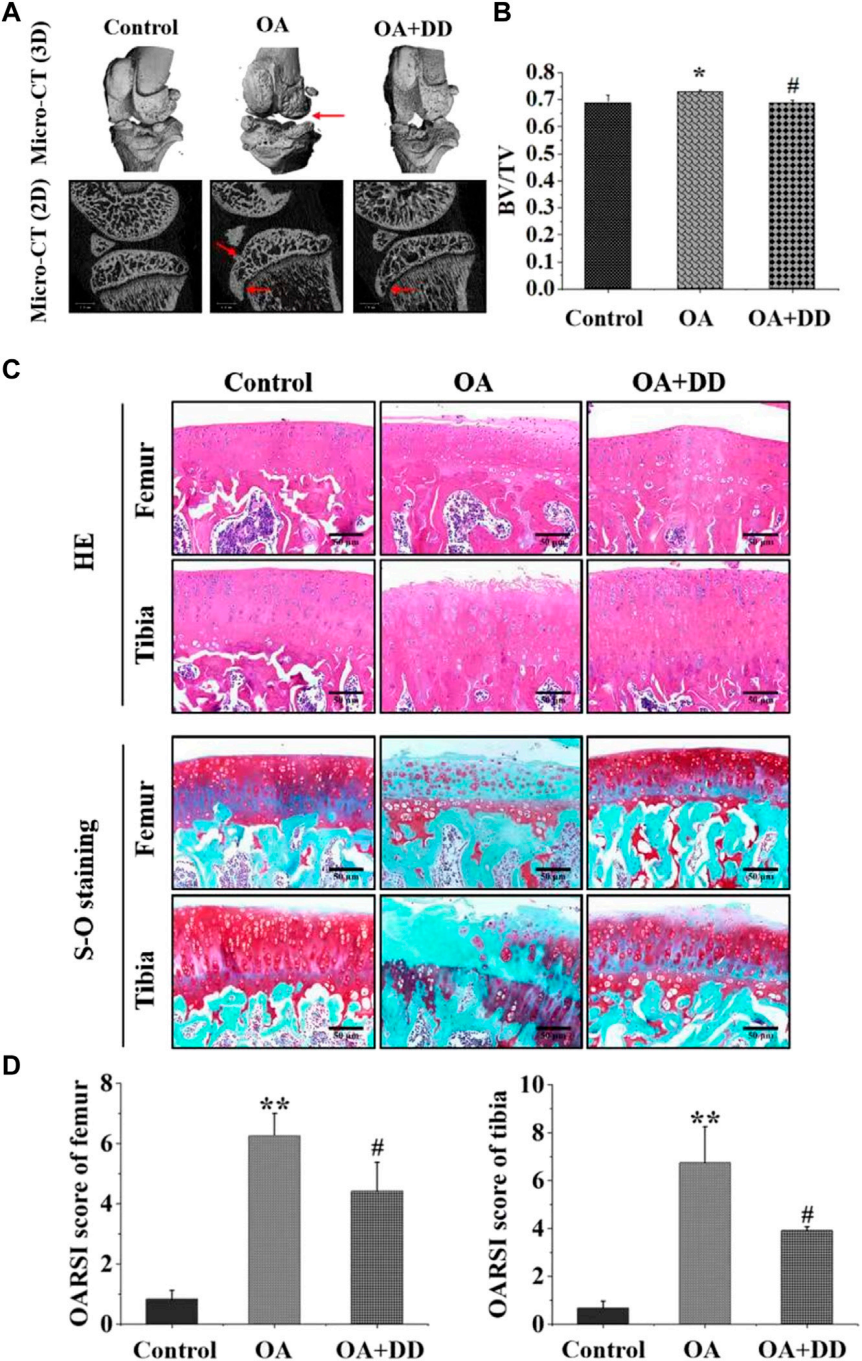
DD alleviated tibial subchondral bone and Cartilage damage. **(A)** Micro-CT was used to analyze 3D images and 2D representative images of DD treated the OA rats, with arrows showing the medial side of the tibial plateau. **(B)** Quantitative micro-CT analysis of tibial subchondral bone with trabecular bone volume per total volume. **(C, D)** Representative images of the knee joint stained with HE staining and S-O staining and OARSI scores after 6 weeks of treatment. The data are presented as means ± SD (n = 3). *p < 0.05, **p < 0.01 compared with the control group; ^#^ *p* < 0.05 compared with the OA group.

Subchondral bone and articular cartilage make up a functional joint unit, which plays a complementary role in the weight-bearing capacity of the joint (Pan et al., 2009). To explore the effects of DD on the tibia subchondral bone of OA rats, the 2D images and reconstructed 3D images obtained from the Micro-CT were used to assess the microarchitecture of the rat subchondral bone. As revealed in Figure 1A, the tibial subchondral bone showed significant changes between control and OA groups, which displayed osteophyte development and the hardened subchondral bone, resulting in cartilage damage in the OA rats. Meanwhile, we observed that DD treatment markedly alleviated the destruction of the tibial subchondral bone. As revealed in Figure 1B, significant differences were observed in the BV/TV of the tibial subchondral bone between the control (0.691 ± 0.027) (*p* < 0.05) and OA group (0.727 ± 0.005). The average value of BV/TV was 0.693 ± 0.009 in DD-treated group, which was lower than that of OA group (0.727 ± 0.005) (*p* < 0.05). These results show that DD treatment prominently alleviated tibial subchondral bone damage relative to the OA group.

To explore the effects of DD on the articular cartilage of OA group, histological analysis of OA was conducted by S-O and HE staining. As shown in Figure 1C, HE staining showed that the OA group exhibited a reduction in chondrocytes and articular cartilage thickness with an irregular morphological structure, while articular cartilage in the control and DD-treated group had a regular morphological structure. Meanwhile, S-O staining showed decreased proteoglycans and stromal disturbances in OA group (Figure 1C). By contrast, the matrix degradation in the DD-treated group was less than in the OA group. This was further confirmed by OARSI scores, which was significantly decreased in the DD-treated group compared with the OA group (Figure 1D). Taken together, these results reinforced the protective effect of DD on OA by alleviating cartilage damage in the rat OA models.

**FIGURE 2:**
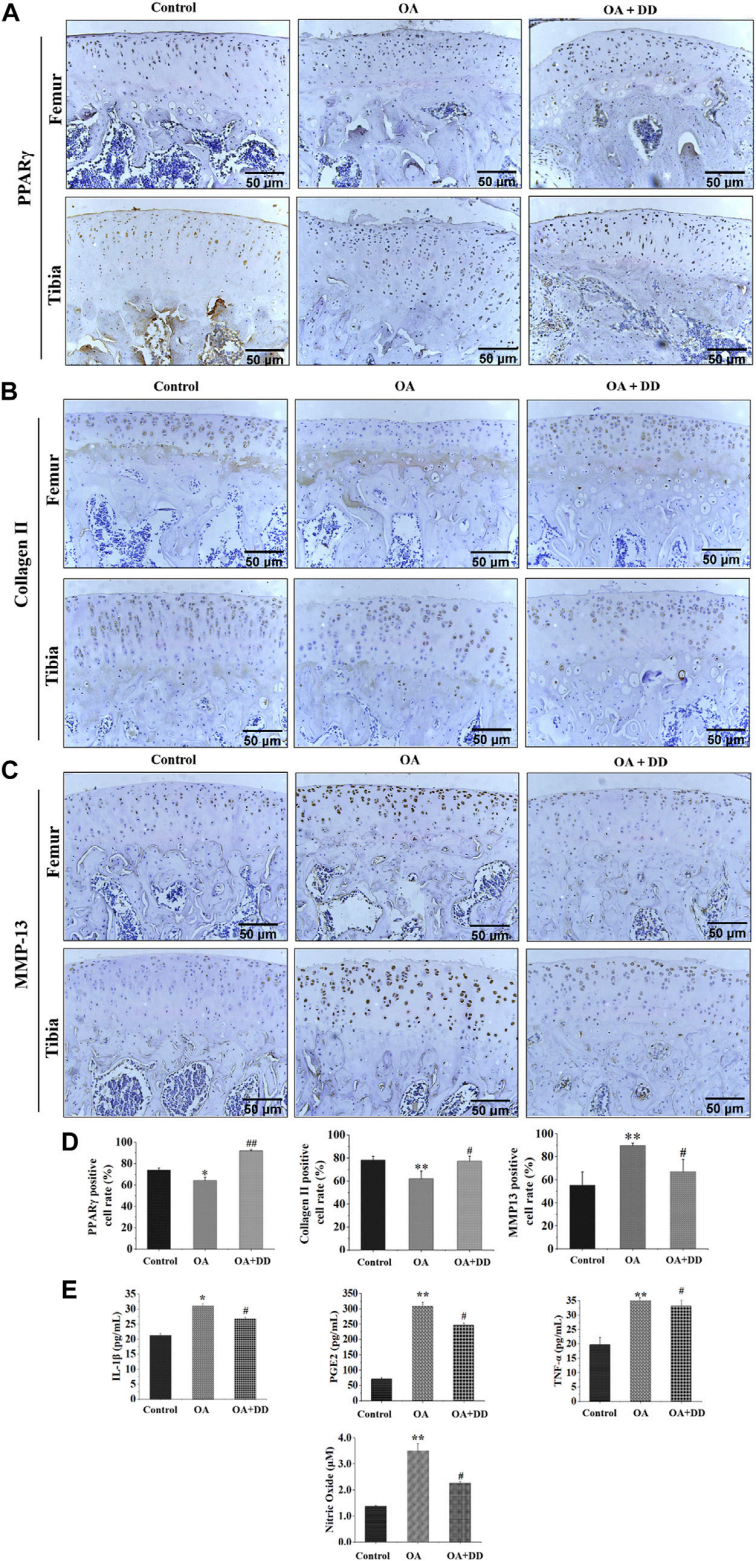
DD reduced cartilage ECM degradation and inhibited inflammatory factor production in OA rats. **(A)** The expression of PPARγ in sections of cartilage; **(B)** The expression of Collage II in sections of cartilage; **(C)** The expression of MMP-13 in sections of cartilage; **(D)** Quantification of histology positive cells of PPARγ, Collage II, and MMP-13; **(E)** The effect of DD on IL-1β, PGE2, TNF-α, and NO inflammatory cytokines. The data are presented as mean ± SD (*n* = 3). *p < 0.05, **p < 0.01 compared with the control group; ^#^ *p* < 0.05, ^##^ *p* < 0.01 compared with the OA group.

PPARγ affects inflammation and extracellular matrix remodeling in OA by regulating transcriptional activity (Afif et al., 2007). We found that the rat OA models demonstrated decreased expression of PPARγ in the articular cartilage. DD inhibited the reduction in PPARγ expression in chondrocytes of OA model rats. MMP-13 and collagen II are key proteins for assessing OA chondrocyte matrix degradation (Wang et al., 2013; Tiku and Madhan, 2016). These results suggest that DD promotes OA matrix formation and inhibits its degradation (Figures 2A–D). Furthermore, we detected the expression of inflammatory cytokines (IL-1β, PGE2, NO, and TNF-α) in the serum by ELISA. As shown in Figure 2E, the production of IL-1β, PGE2, NO, and TNF-α increased significantly in the OA group. In DD-treated group, the release of IL-1β, PGE2, NO, and TNF-α were decreased sharply in the serum. These results demonstrate that DD inhibited the reduction of PPARγ in OA chondrocytes and alleviate cartilage degeneration and inflammation.

### Virtual screening of potentially active constituents targeting DNMT1 from DD

DNMT1-mediated hypermethylation of PPARγ promoter in chondrocytes is a key mechanism for promoting OA (Ball et al., 2022). To identify the active constituents of DD for the treatment of OA by targeting DNMT1, 906 constituents from DD were docked into the pocket for DNMT1. In this study, the constituents with a docking score ≤ −5 kcal/mol were defined as potentially active constituents (Kun-Yi et al., 2013). The total number of constituents with potential DNMT1 inhibitory activities was 608 in DD (Supplementary Table S1). Each herb had many potential inhibitory constituents (Supplementary Table S1). Therefore, according to the Pharmacopoeia of the People’s Republic of China (2020) and the literature of on herb main constituents, seven constituents, including isoliquiritigenin, emodin, taxifolin, catalpol, alisol A, zingerone, and schisandrin C which showed high docking scores were considered for further analysis (Table 1).

**TABLE 1:** Information of the seven potential active constituents.

Constituent	Herb	Docking score (kcal/mol)
Isoliquiritigenin	Glycyrrhizae Radix et Rhizoma	−8.66
Emodin	Alismatis Rhizoma	−8.02
Taxifolin	Cinnamomi Ramulus	−7.87
Catalpol	Rehmanniae Radix Praeparata	−6.84
Alisol A	Alismatis Rhizoma	−6.56
Zingerone	Zingiberis Rhizoma/Schisandrae Chinensis Fructus	−6.44
Schisandrin C	Schisandrae Chinensis Fructus	−6.36

### Effect of potentially active constituents on the viability of chondrocytes

**FIGURE 3:**
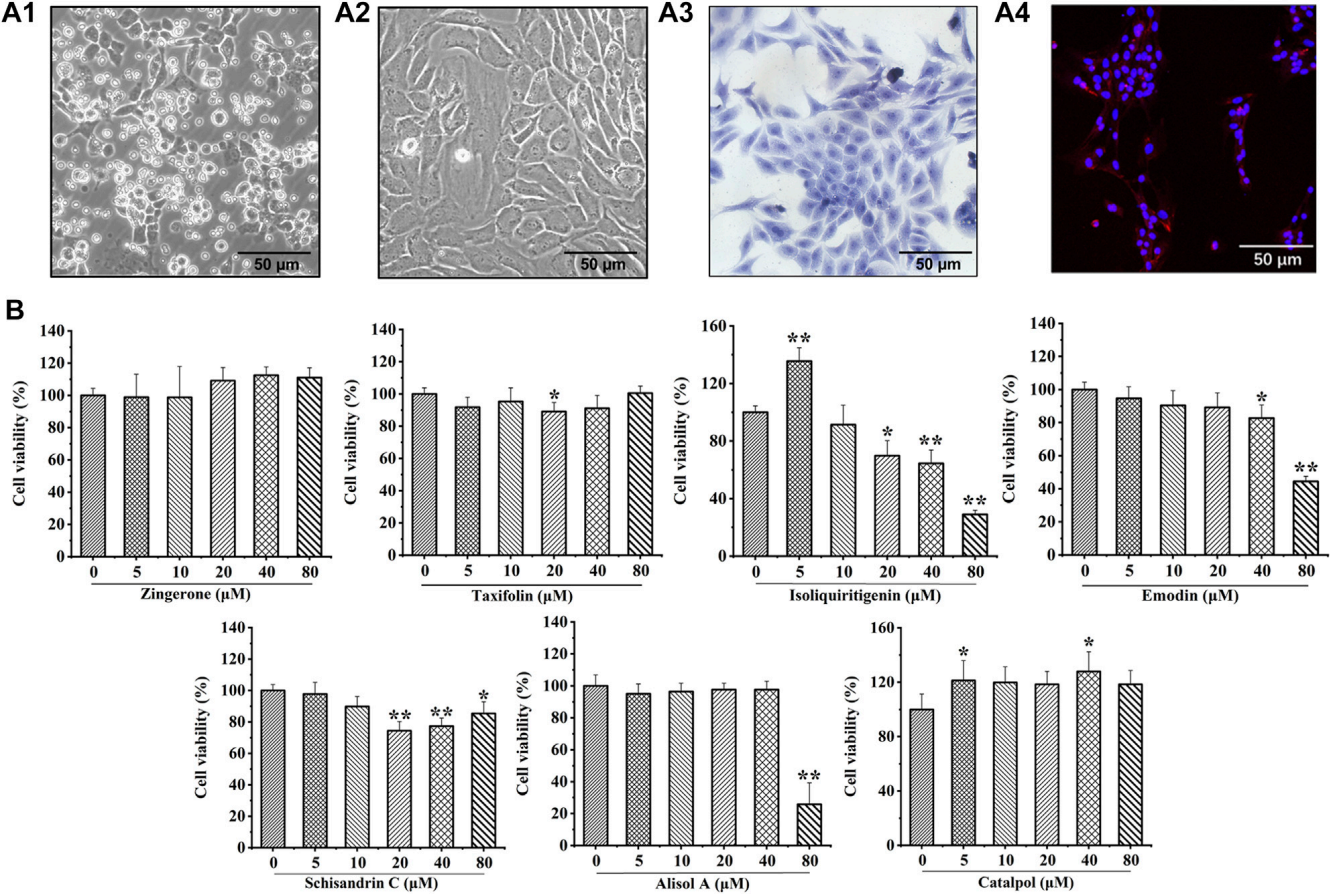
Culture and identification of rat chondrocytes, and the effects of seven potential active constituents (Isoliquiritigenin, Emodin, Taxifolin, Catalpol, Alisol A, Zingerone, and Schisandrin C) on rat chondrocytes viability. **(A1)** Primary rat chondrocytes cultured for 24 h; **(A2)** Primary rat chondrocytes cultured for 7 days; **(A3)** Toluidine blue staining of primary rat chondrocytes; **(A4)** Immunofluorescence staining of collagen II of primary rat chondrocytes; **(B)** Effect of seven potential active constituents (Isoliquiritigenin, Emodin, Taxifolin, Catalpol, Alisol A, Zingerone, and Schisandrin C) on chondrocytes viability at different concentrations (0, 5, 10, 20, 40, and 80 μM) for 24 h. *p < 0.05, **p < 0.01 compared with the control group.

Chondrocytes were extracted from 3-week-old SD rats. Newly isolated chondrocytes appeared round, whereas most cells cultured for 24 h showed adherent growth. At 7 days of cell culture, chondrocytes showed uniform polygonal or short spindle shapes microscopically (Figure 3A1, A2). Toluidine blue staining and collagen II immunofluorescence staining were utilized to identify primary rat chondrocytes (Figure 3A3, A4).

The cytotoxic effect of isoliquiritigenin, emodin, taxifolin, catalpol, alisol A, zingerone, and schisandrin C were evaluated after treatment with different concentrations (0, 5, 10, 20, 40, and 80 μM) for 24 h using a CCK-8 assay. As shown in Figure 3B, isoliquiritigenin (10 µM), emodin (20 µM), taxifolin (10 µM), catalpol (10 µM), alisol A (20 µM), zingerone (20 µM), and schisandrin C (10 µM) did not affect the viability of chondrocytes. Hence, these concentrations of potential active constituents were chosen for subsequent experiments.

### Effects of potentially active constituents on PPARγ expression

**FIGURE 4:**
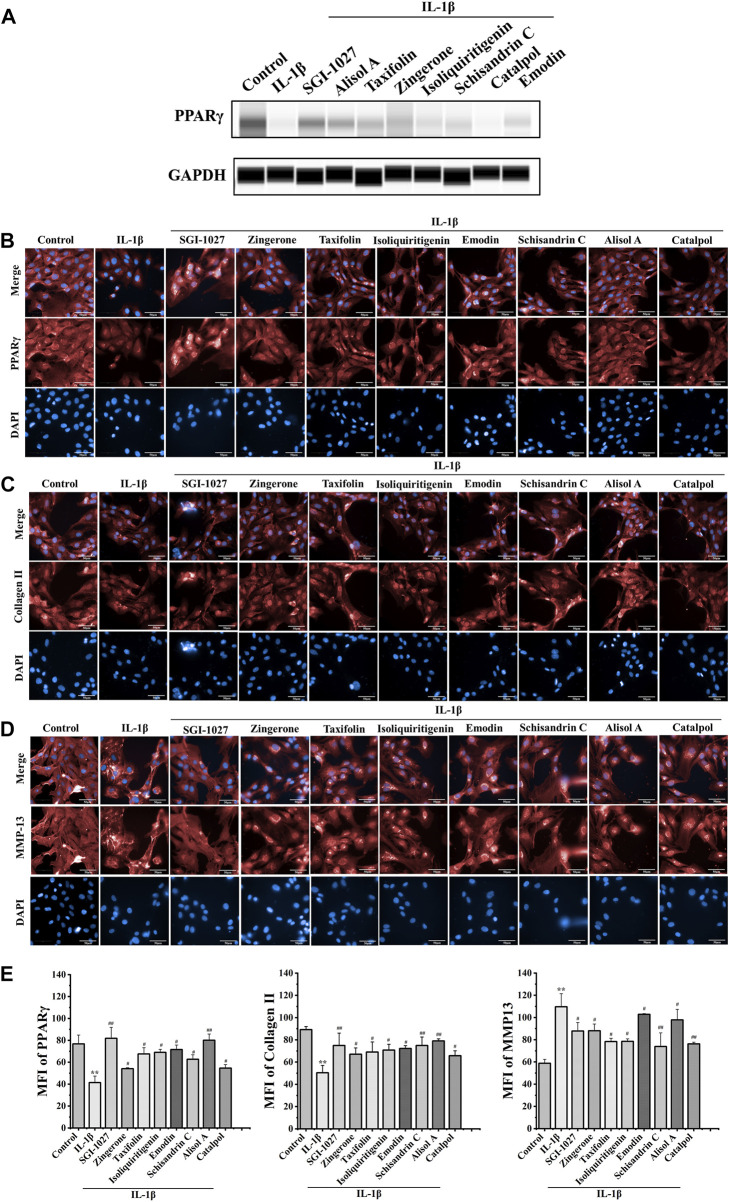
Effect of seven potential active constituents (Isoliquiritigenin, Emodin, Taxifolin, Catalpol, Alisol A, Zingerone, and Schisandrin C) on PPARγ, collagen II, and MMP-13 in IL-1β-treated chondrocytes. **(A)** The expression of PPARγ in IL-1β-treated chondrocytes by protein capillary electrophoresis; **(B)** PPARγ (red) was detected by immunofluorescence; **(C)** Collagen II (red) was detected by immunofluorescence; **(D)** MMP-13 (red) was detected by immunofluorescence; **(E)** Quantification analysis of the immunofluorescence of PPARγ, collagen II, and MMP-13. **p < 0.01 compared with the control group; ^#^ *p* < 0.05, ^##^ *p* < 0.01 compared with the IL-1β group.

The results of *in vivo* experiments confirmed that DD can promote the expression of PPARγ in OA chondrocytes. To explore the effect of above seven potential active constituents on PPARγ expression, we used protein capillary electrophoresis to test the expression of PPARγ in chondrocytes after treatment with IL-1β. As shown in Figure 4A, most of the seven constituents reversed the decline of PPARγ in chondrocytes IL-1β-treated.

### Effects of potentially active constituents on of PPARγ, collagen II, and MMP-13 in IL-1β-treated chondrocytes

PPARγ plays a role in the regulation of matrix decomposition (Afif et al., 2007). Previous reports showed that collagen II is a major component of the cartilage matrix (Handa et al., 2021). MMP-13 can hydrolyze collagen II, which is considered as a significant biomarker to assess OA therapeutic effects and OA progression (Corciulo et al., 2017). To further verify the effect of DD active constituents on PPARγ expression in chondrocytes, we measured the extent to which the active constituents promoted PPARγ expression in IL-1β-induced chondrocytes by a high content-laser confocal system (Figures 4B,E). These potential active constituents of DD could also inhibit IL-1β-induced downregulation of collagen II and the production of MMP-13 (Figures 4C,D,E). Based on the experimental results of the above methods, five active constituents, including alisol A, emodin, taxifolin, isoliquiritigenin, and schisandrin C inhibited the changes of these proteins in IL-1β-treated chondrocytes. These five active constituents should be the representative active compounds in DD which can ameliorate OA *via* PPARγ preservation.

### Effects of potentially active constituents on TNF-α, NO, and PGE2 production in IL-1β-treated chondrocytes

**FIGURE 5:**
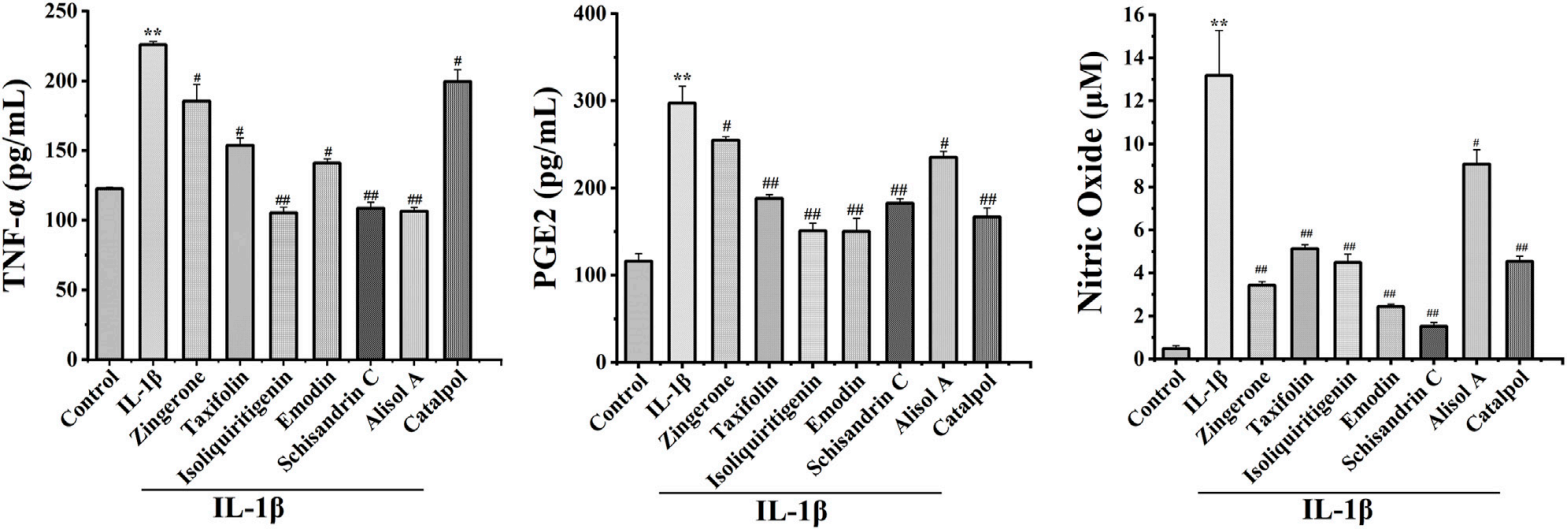
Effects of seven potential active constituents (Isoliquiritigenin, Emodin, Taxifolin, Catalpol, Alisol A, Zingerone, and Schisandrin C) on IL-1β-induced PGE2, TNF-α, and NO synthesis in rat chondrocytes. The culture supernatants were then harvested for further detection. **p < 0.01 compared with the control group; ^#^ *p* < 0.05, ^##^ *p* < 0.01 compared with the IL-1β group.

Inflammation is causally involved in OA pathogenesis (Kraus et al., 2015). To further analyze the anti-inflammatory effects of active constituents, we measured the levels of major inflammatory mediators TNF-α, NO, and PGE2 in cell culture media. The active constituents decrease TNF-α, NO, and PGE2 secretion in IL-1β-treated chondrocytes. These results demonstrated that active constituents of DD mentioned above have the effect of inhibiting IL-1β-induced inflammation (Figure 5).

### Effects of potentially active constituents on rat cartilage damage in IL-1β-treated rat cartilage explants

**FIGURE 6:**
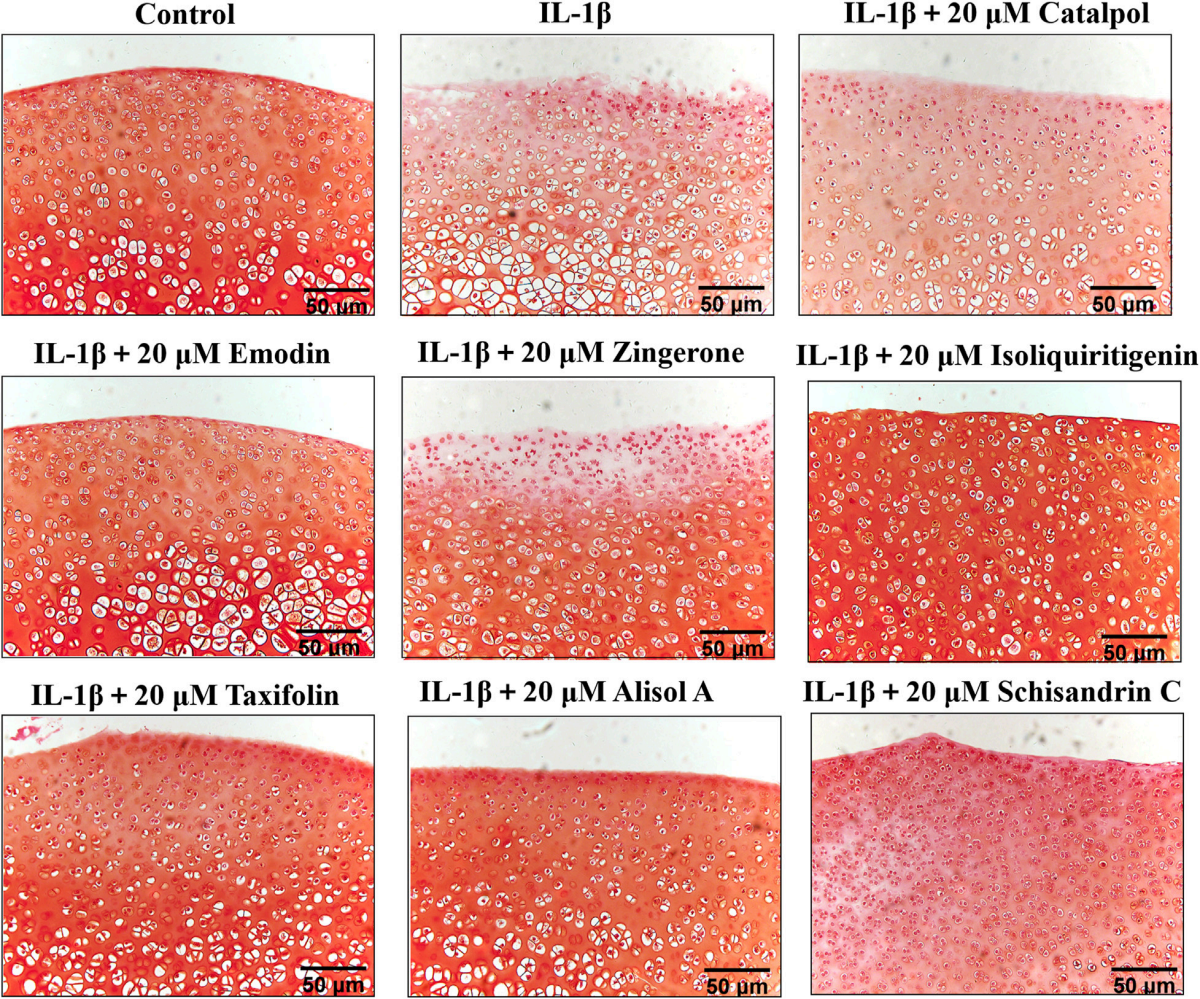
Rat cartilage explants were exposed to 20 μM active constituents with IL-1β (10 ng/ml) for 3 days and stained with Safranin O staining.

To manifest the effects of active constituents on IL-1β-treated rat cartilage explants, an *ex vivo* model culture of rat cartilage explants was established (Tu et al., 2019b). Active constituents at 20 μM were selected for cartilage explants intervention. The cartilage matrix degradation was assessed by safranin O staining analysis of samples cultured for 3 days with or without IL-1β plus compound treatment. Compared with the control group, IL-1β treatment of explants enhanced matrix degradation of the cartilage explants (Figure 6). The active constituents in DD alleviated this change compared with the IL-1β-treated alone rat cartilage explants. In the alisol A, isoliquiritigenin, and emodin intervention groups, cartilage explants matrix degradation was alleviated. The extent of cartilage damage was assessed by HE analysis has a similar trend (Supplementary Figure S4, Supplementary Figure S5). These results suggested that the five constituents, such as alisol A, emodin, taxifolin, isoliquiritigenin, and schisandrin C mitigated matrix degradation and cartilage damage in rat cartilage explants.

### Molecular dynamic simulation and binding interaction pattern analysis

**FIGURE 7:**
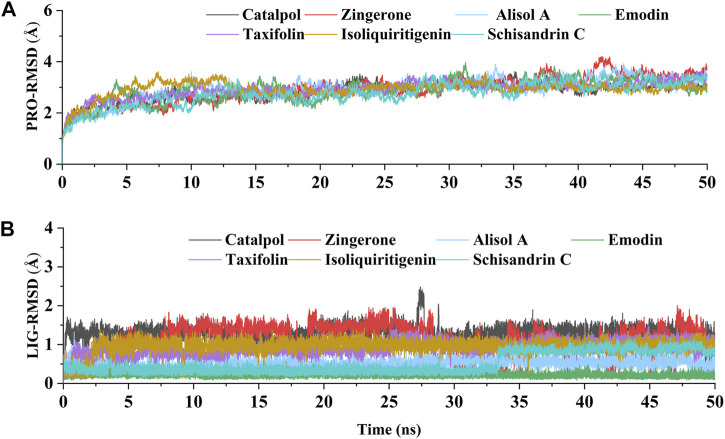
The RMSD values relative to the initial structure. **(A)** RMSD of the backbone atoms of DNMT1 in each constituents-DNMT1 complex; **(B)** RMSD of the heavy atoms of seven potential active constituents in each constituents-DNMT1 complex.

The above experimental results show that the active constituents of DD appear to be able to ameliorate osteoarthritis by targeting DNMT1 to preserve PPARγ expression. To further study the interaction of DNMT1-isoliquiritigenin, DNMT1-emodin, DNMT1-taxifolin, DNMT1-catalpol, DNMT1-alisol A, DNMT1-zingerone, and DNMT1-schisandrin C complexes at the molecular level, molecular dynamics simulations with the MM-GBSA method was applied. After acquiring the trajectory of molecular dynamic simulation, the structural stability of all complexes in 50 ns simulation were evaluated by root means square deviation (RMSD) values of Cα atoms of protein and heavy atoms of ligand. As Figures 7A,B showed that all the simulations were well equilibrated during 50 ns and the ligand did not undergo a remarkable conformational change.

For most enzyme systems, the bioactivities of inhibitors are largely related to binding affinities (Wang et al., 2019). In this study, the binding free energies between the seven potential active constituents (isoliquiritigenin, emodin, taxifolin, catalpol, alisol A, zingerone, and schisandrin C) and DNMT1 complexes were calculated by the MM-GBSA method, and the results were showed that all seven constituents have good binding affinity (Table 2). Thereinto, the constituents with relatively good binding free energies are alisol A (-40.05 kcal/mol), emodin (−27.56 kcal/mol), taxifolin (−27.35 kcal/mol), isoliquiritigenin (−20.61 kcal/mol), and schisandrin C (−25.18 kcal/mol). Preliminary results showed that, alisol A, emodin, taxifolin, isoliquiritigenin, and schisandrin C achieved a stable binding with DNMT1, which was highly consistent with the results of cell and explant experiments.

**TABLE2:** The average of binding free energy of DNMT1 complex obtained using Amber20 (kcal/mol).

Complex	Energy Component				
	ΔE_vdw_	ΔE_ele_	ΔG_gas_	ΔG_solv_	ΔG_bind_
DNMT1-Catalpol	−28.31	−20.02	−48.33	34.08	−14.25
DNMT1-Zingerone	−18.46	−9.43	−27.88	14.09	−13.80
DNMT1-Alisol A	−43.94	−44.50	−88.44	48.39	−40.05
DNMT1-Emodin	−35.42	−21.25	−56.68	29.12	−27.56
DNMT1-Taxifolin	−25.82	−61.06	−86.88	59.52	−27.35
DNMT1-Isoliquiritigenin	−30.19	−39.22	−69.41	48.79	−20.61
DNMT1-Schisandrin C	−38.53	−7.96	−46.49	21.31	−25.18

**FIGURE 8:**
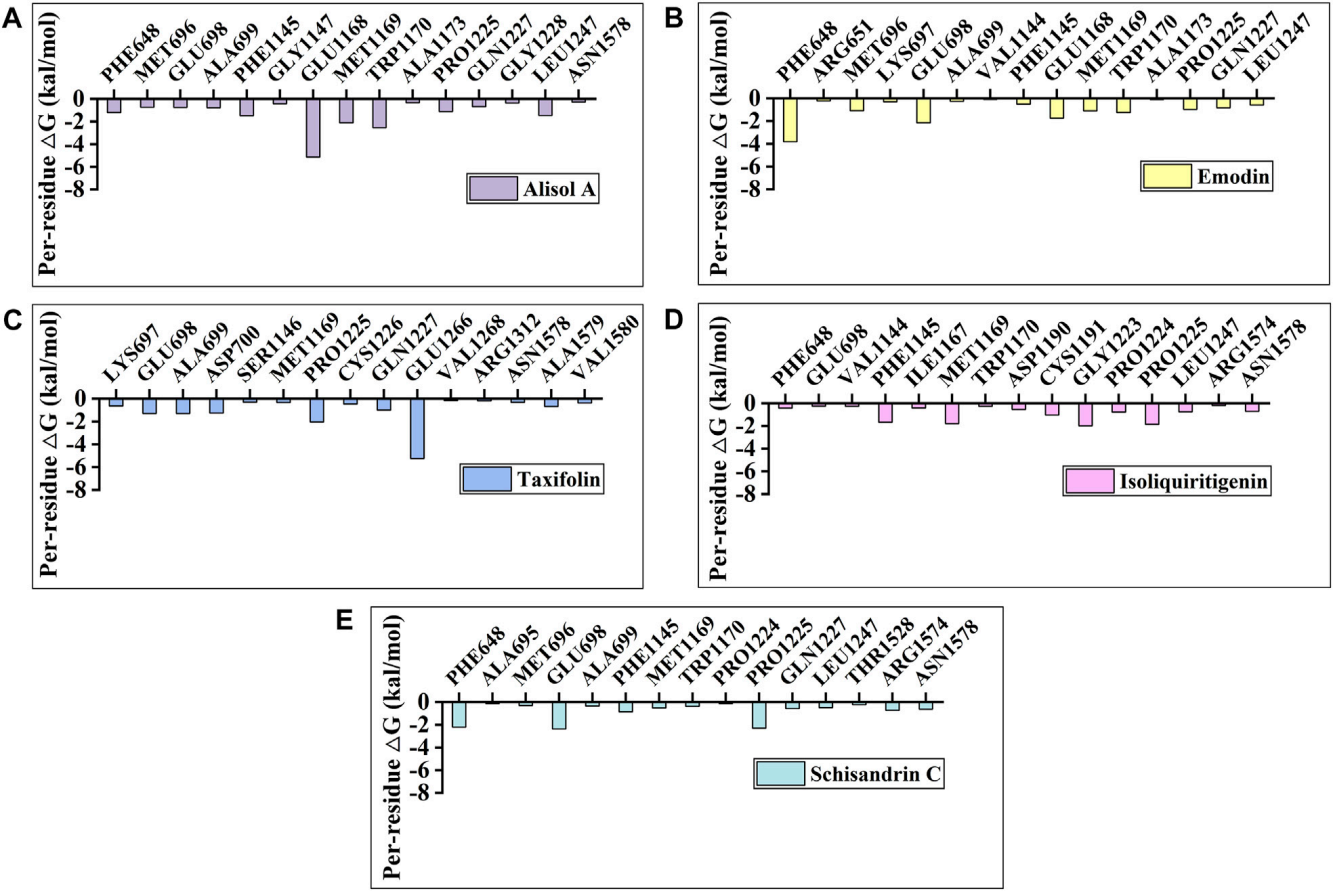
Per-residue binding free energy decomposition of **(A)** Alisol A; **(B)** Emodin; **(C)** Taxifolin; **(D)** Isoliquiritigenin; and **(E)** Schisandrin C binding to DNMT1.

**FIGURE 9:**
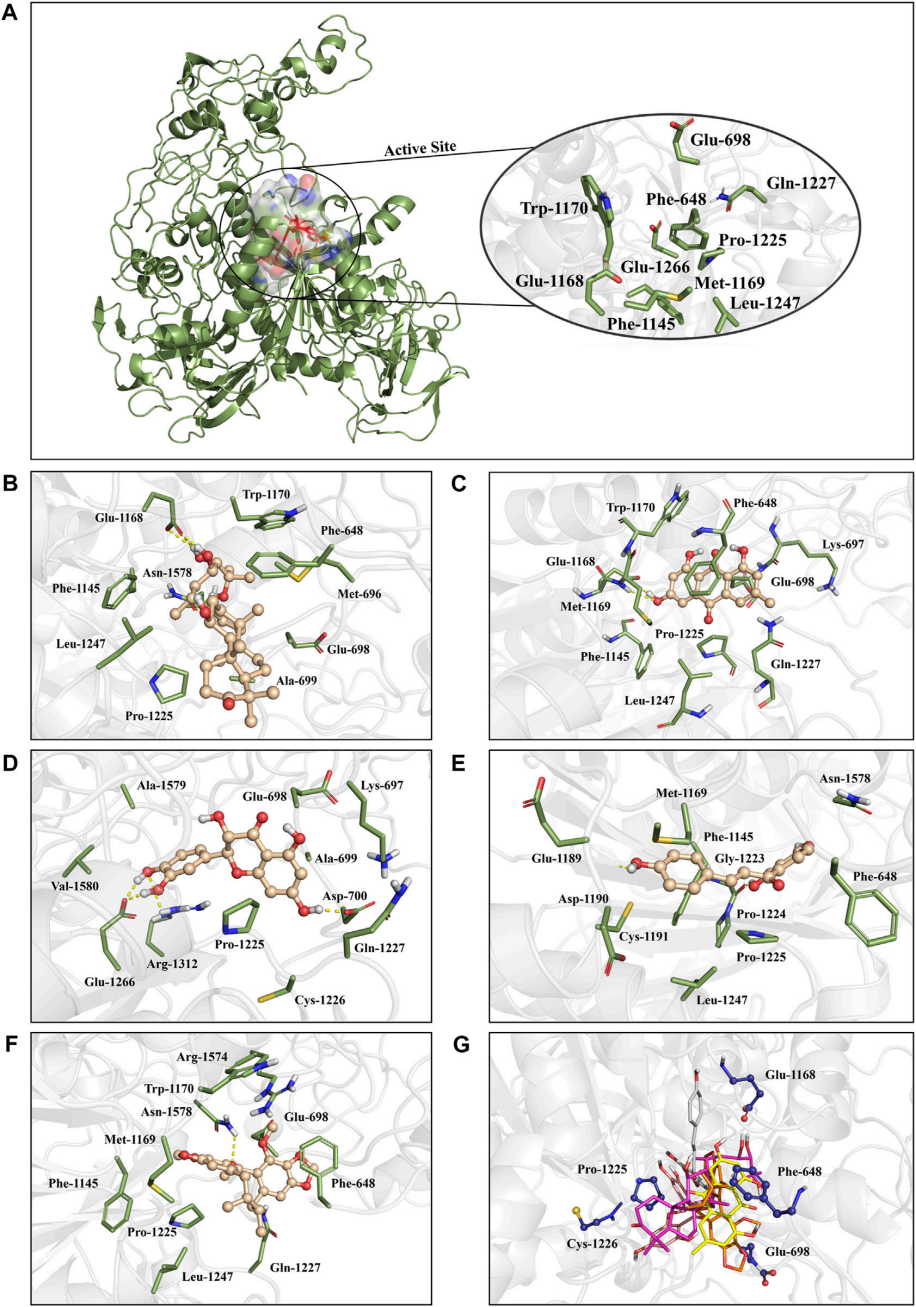
The positions of the active constituents are anchored by the interaction with DNMT1. **(A)** The complex structure and active site of DNMT1. The active site of DNMT1 was shown in rainbow surface. **(B)** Interaction mode of alisol A with the DNMT1; **(C)** Interaction mode of emodin with the DNMT1; **(D)** Interaction mode of taxifolin with the DNMT1; **(E)** Interaction mode of isoliquiritigenin with the DNMT1; **(F)** Interaction mode of schisandrin C; and **(G)** above five constituents with the DNMT1.

To identify the key amino acids for the protein-ligand interaction, residue energy decomposition of the binding free energies was calculated and the binding modes of all above complex were investigated by the representative conformations extracted from the MD trajectories. In this study, the total binding free energy was decomposed and the top 15 amino acids with the largest contributions to ligand binding were recorded (Figure 8). The results showed that Phe 648, Glu 698, and Glu 1,168 are the important amino acids in the DNMT1-alisol A complex system (Figure 8A), and a hydrogen bond was found to be formed between alisol A and amino acids Glu 1,168 (Figures 9A,B). Phe 648, Glu 698, and Glu 1,168 were the important amino acids in the DNMT1-emodin complex system, and the hydrogen bond was found to be formed between emodin and amino acids Glu 1,168 and Met 1,169 (Figure 8B, Figure 9C). In the DNMT1-taxifolin complex system, Pro 1,225 and Glu 1,266 were the important amino acids, and a hydrophobic were formed between Glu 1,266, Arg 1,312, and taxifolin (Figure 8C, Figure 9D). Interestingly, the active residues here, such as Met 1,169, Glu 1,266, Glu 1,168, and Pro 1,225, are the same as those reported by Liang et al. (Liang et al., 2020). It is indicated that the binding pockets of above-mentioned active constituents have partial overlap with the allosteric pockets (Zhu et al., 2021). Phe 1,145, Met 1,169, Gly 1,223, and Pro 1,225 were the important amino acids in the DNMT1-isoliquiritigenin complex system (Figure 8D, Figure 9E). In the DNMT1-schisandrin C complex systems, Phe 648, Glu 698, and Pro 1,225 were the important amino acids (Figure 8E, Figure 9F). Targeted hot spot amino acids common to the above constituents are Phe 648, Glu 698, and Glu 1,168 might were key interacting amino acids for binding of DNMT1 inhibitors. Besides, the five constituents, such as alisol A, emodin, taxifolin, isoliquiritigenin, and schisandrin C had similar combined amino acids and binding patterns (Figure 9G).

Based on the calculation results, alisol A may have a strong targeted inhibition effect with a relatively high binding free energy (-40.05 kcal/mol), which showed a similar binding energy as a DNMT1 inhibitor DC-05 (Xie et al., 2019) (Tu et al., 2019b). Alisol A also had a significant promoted effect on PPARγ expression in IL-1β-treated chondrocytes by protein capillary electrophoresis and immunofluorescence assays. To assess whether alisol A can reverse PPARγ suppression by targeting DNMT1, the effect of alisol A on methylation of PPARγ promoter was conducted by MSP method. The results show that alisol A can reverse the methylation of PPARγ promoter, which was similar to that of DNMT1 inhibitor SGI-1027 (Figure S3A-B) (Zhu et al., 2019). These results suggested that alisol A could be developed as a natural epigenetic regulator derived from Chinese herbal formula.

## Discussion

Inflammation-induced destruction of articular cartilage is the most prominent feature of OA (Kraus et al., 2015; Quicke et al., 2022). At present, the treatment of OA is mainly to relieve pain, and there is a lack of targeted drugs to slow down the inflammatory destruction of cartilage (Goldring and Otero, 2011). The destruction of articular cartilage is closely related to the degradation of the cartilage matrix induced by inflammation (Goldring and Otero, 2011; Chen et al., 2021b), and TCM has an original theoretical system for maintaining the physiological stability of bone (Sun et al., 2022). In this study, we first confirmed by *in vivo* experiments that DD reduced papain-induced osteochondral injury and significantly decreased chondrocyte matrix degradation. Therefore, our study indicates that DD exerts its protective effect on cartilage mainly by decreasing the degradation of the osteochondral matrix.

Recent studies found that PPARγ played a key role in the degradation and protection of osteochondral matrix in OA (Afif et al., 2007; Fahmi et al., 2011). We also found that PPARγ expression decreased in the osteochondral matrix in OA model rats, while DD increased the expression of PPARγ. The hypermethylation of PPARγ promoter is one of the reasons for the decrease of PPARγ in OA (Zhu et al., 2019). Therefore, DD is likely to participate in the methylation regulation of PPARγ promoter and preserve the expression of PPARγ. The results of molecular docking suggest that isoliquiritigenin, emodin, taxifolin, catalpol, alisol A, zingerone, and schisandrin C in DD have high docking affinity to DNMT1, and should be the main potentially active constituents to preserve the expression of PPARγ in DD (Chen et al., 2021a).

The seven identified active constituents have been identified as the main constituents of DD that target DNMT1. Specifically, alisol A, emodin, taxifolin, isoliquiritigenin, and schisandrin C promoted PPARγ expression and cartilage matrix formation. The prominent roles of these constituents in promoting cartilage matrix formation are consistent with the *in vivo* experiment of DD. It was further confirmed that these active constituents showed protective effect against IL-1β-induced inflammatory injury by culturing rat cartilage explants. Among them, alisol A and emodin could effectively mitigate cartilage damage. Finally, through molecular dynamics simulations with the MM-GBSA method, alisol A, emodin, taxifolin, isoliquiritigenin, and schisandrin C were found to achieve a stable binding pattern. The common targeted hot spot residues of the above constituents were Phe 648, Glu 698, and Glu 1,168. Therefore, alisol A with a relatively high binding free energy was considered a key constituent in DD to reverse PPARγ demethylation by targeting DNMT1 inhibition.

In conclusion, in this work, combined with *in vivo* experiments and virtual screening, cell and explant experiments, the protective effect of DD on OA and the active constituents maintaining matrix stability of cartilage through epigenetic regulation of PPARγ have been systematically elucidated. The five active constituents consist, such as alisol A, emodin, taxifolin, isoliquiritigenin, and schisandrin C in DD could promote the expression of PPARγ and the formation of cartilage matrix. Moreover, molecular dynamics simulations with MM-GBSA method indicated the five active constituents also achieved a stable binding with DNMT1, an effective target for regulating the epigenetic expression of PPARγ. This work demonstrates that DD has material basis for its therapeutic role in OA through epigenetic regulation of PPARγ and the active constituents, such as alisol A, deserves further investigation as an epigenetic modulator in the treatment of OA.

## Data Availability

The original contributions presented in the study are included in the article/Supplementary Material, further inquiries can be directed to the corresponding authors.
