# Supplementary material for: Deciphering the active constituents of Dabushen decoction of ameliorating osteoarthritis via PPARγ preservation by targeting DNMT1
Source: Front Pharmacol. 2022 Nov 23;13:993498. doi: 10.3389/fphar.2022.993498 (PMC9727303; doi:10.3389/fphar.2022.993498)
Supplement: Supplementary file 1 [file Table2.docx]

***Supplementary Material***

**Supplementary Figures and Tables**

**Supplementary Figures**


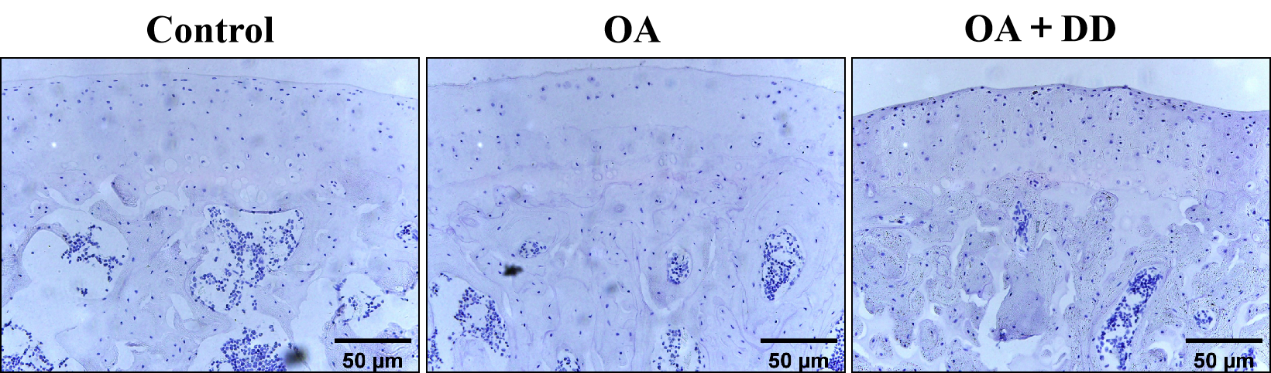


**Supplementary Figure 1.** Immunohistochemistry DAB staining with rat negative control on cartilage. PBS as the primary antibody was used as a negative control.

**Supplementary Figure 2.** The structures of seven potentially active constituents from DD obtained through virtual screening.


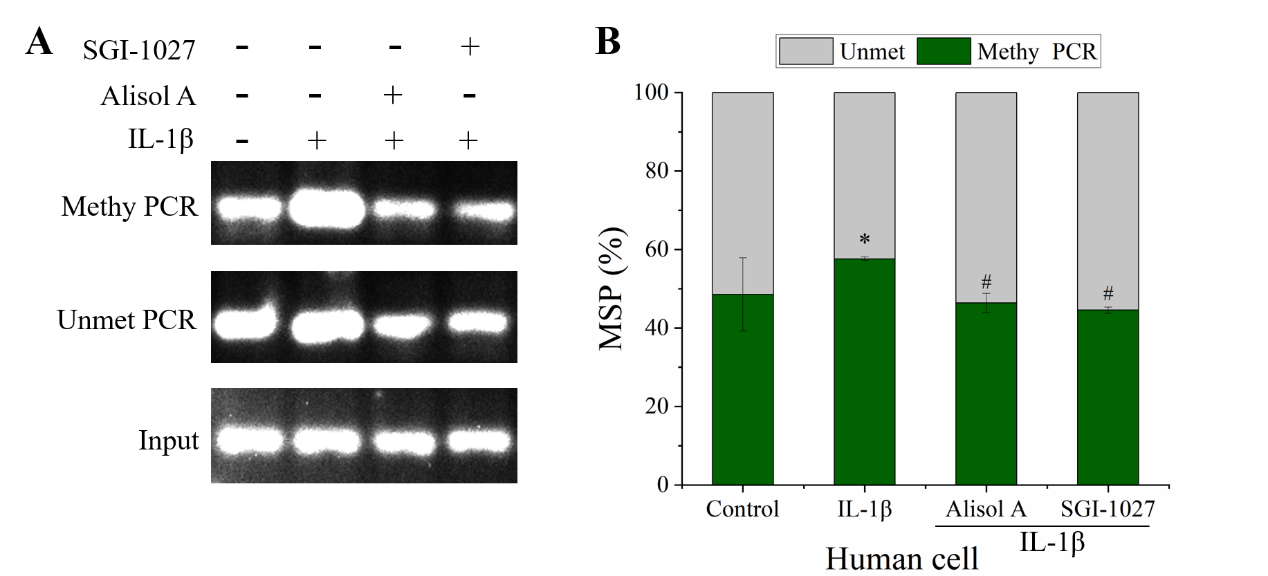


**Supplementary Figure 3.** Methylation profiling of PPARγ promoter by methylation-specific PCR (MSP). (A) Cell MSP analysis. Representative MSP products from cultured human SW1353 chondrocytes treated with IL-1β (10 ng/mL) and Alisol A (20 μM), SGI-1027 for 24 hours were analysed by agarose gel electrophoresis. (B) Quantification of MSP. Values are normalised with input PCR and expressed as mean ± SD of methylation/unmethylated PCR over total PCR products based on three independent experiments. **p*<0.05 compared with the control group; ^#^*p*<0.05 compared with the IL-1β group.


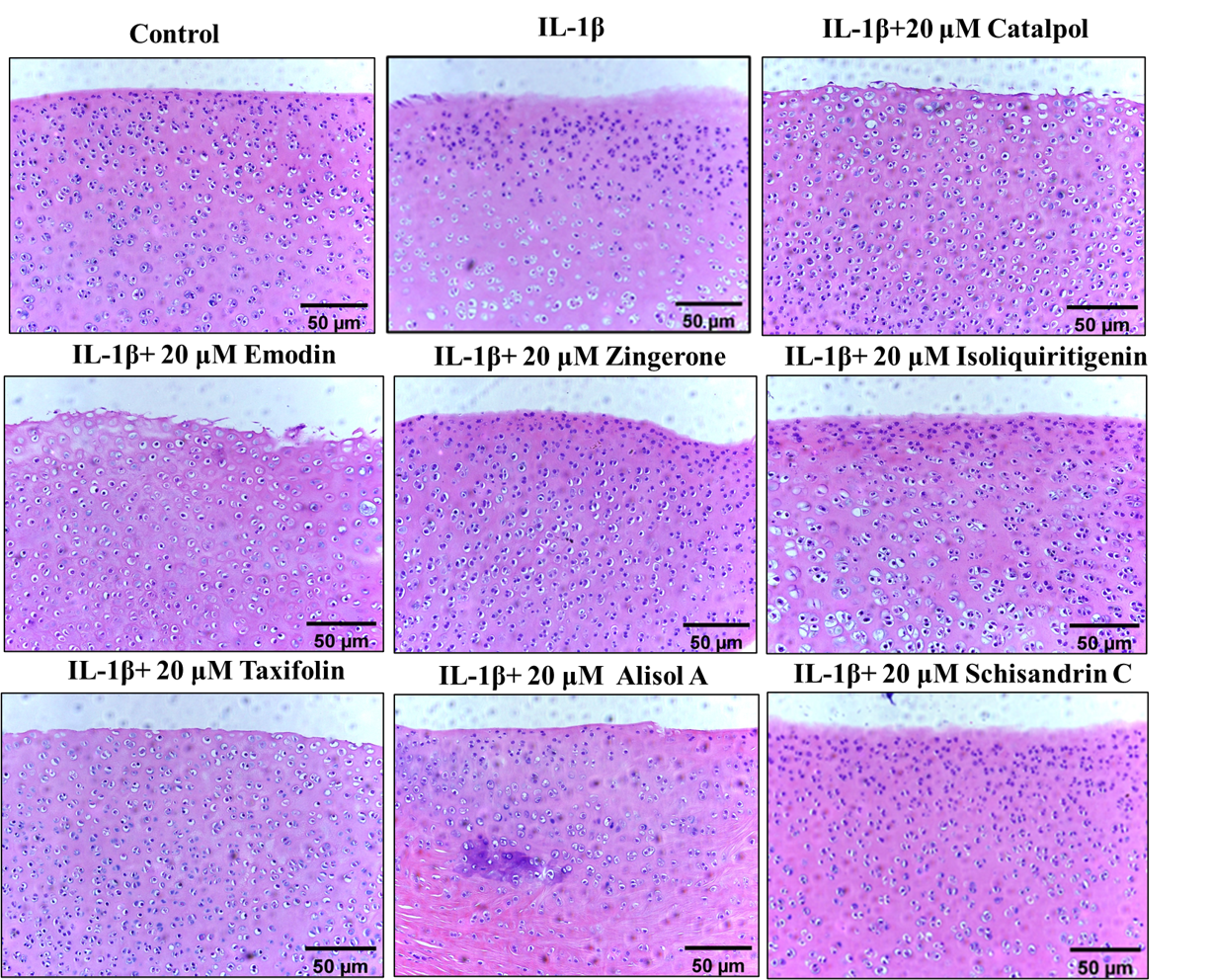


**Supplementary Figure 4**. Rat cartilage explants were exposed to 20 μM active constituents with IL-1β (10 ng/ml) for 3 days and stained with HE.


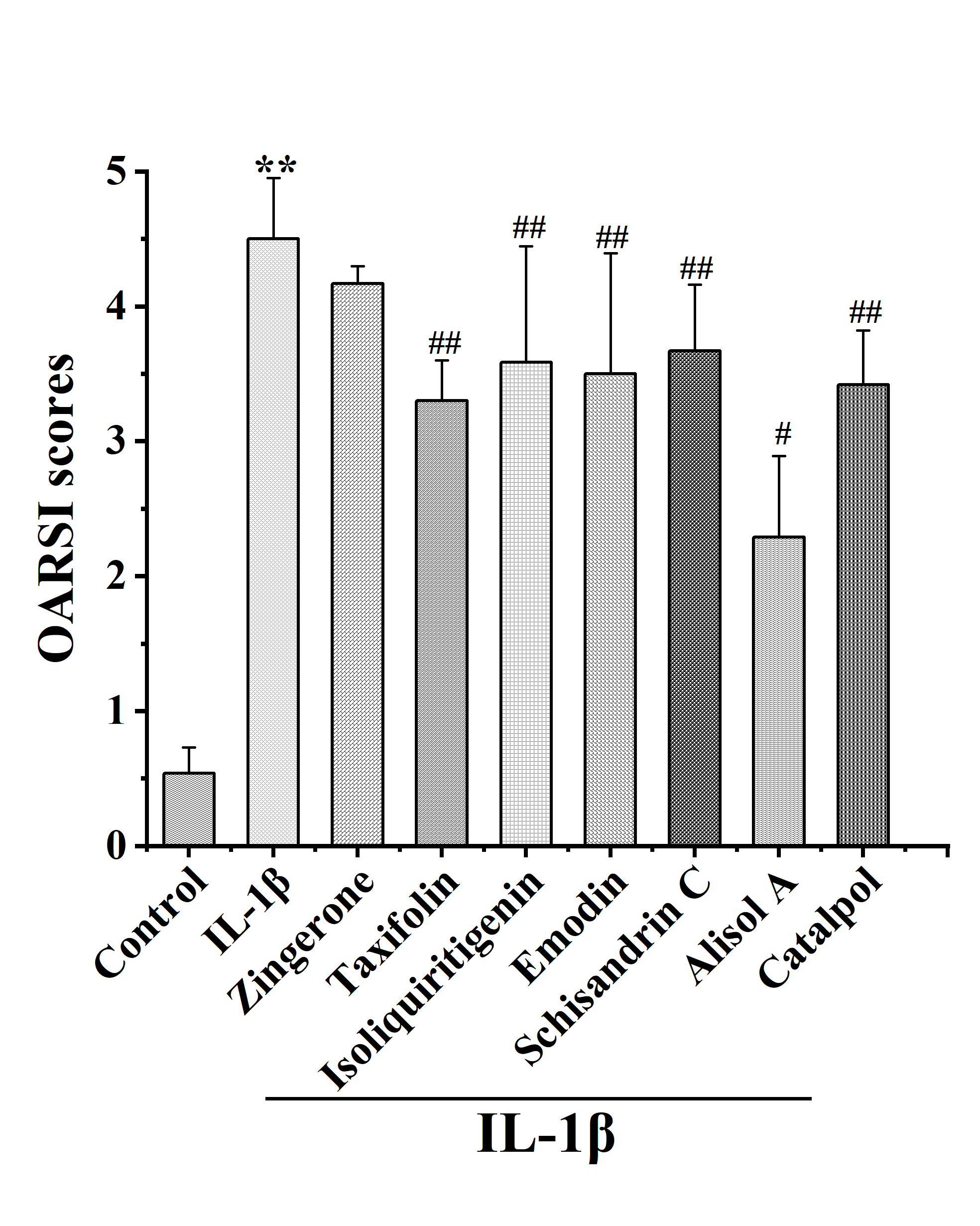


**Supplementary Figure 5**. Rat cartilage explants were exposed to 20 μM Isoliquiritigenin, Emodin, Taxifolin, Catalpol, Alisol A, Zingerone, and Schisandrin C with IL-1β (10 ng/ml) and OARSI scores after for 3 days. The data is presented as means ± SD (n = 6). **p* < 0.05, ***p* < 0.01 compared with the control group; ^#^*p* < 0.05, ^##^*p* < 0.01 compared with the OA group.

**Supplementary Tables**

**Supplementary Table 1.** Number statistics of potentially active constituents from DD obtained through virtual screening targeting DNMT1.

| NO. | Latin name of the herb | The number of compounds | Number of compounds with docking score ≤−5 kcal/mol |
| --- | --- | --- | --- |
| 1 | *Schisandrae Chinensis Fructus* | 126 | 95 |
| 2 | *Glycyrrhizae Radix et Rhizoma* | 272 | 230 |
| 3 | *Zingiberis Rhizoma* | 144 | 69 |
| 4 | *Cinnamomi Ramulus* | 220 | 152 |
| 5 | *Rehmanniae Radix Praeparata* | 74 | 61 |
| 6 | *Alismatis Rhizoma* | 46 | 40 |
| 7 | *Lophatheri Herba* | 4 | 4 |
| Total |  | 794* | 608* |

* The total in the table is the results after deduplication.
